# Supplementary material for: Potential Health Effects of Heavy Metals and Carcinogenic Health Risk Estimation of Pb and Cd Contaminated Eggs from a Closed Gold Mine Area in Northern Thailand
Source: Foods. 2022 Sep 9;11(18):2791. doi: 10.3390/foods11182791 (PMC9498197; doi:10.3390/foods11182791)
Supplement: Supplementary file 1 [file foods-11-02791-s001.zip › foods-1907686-supplementary.pdf]

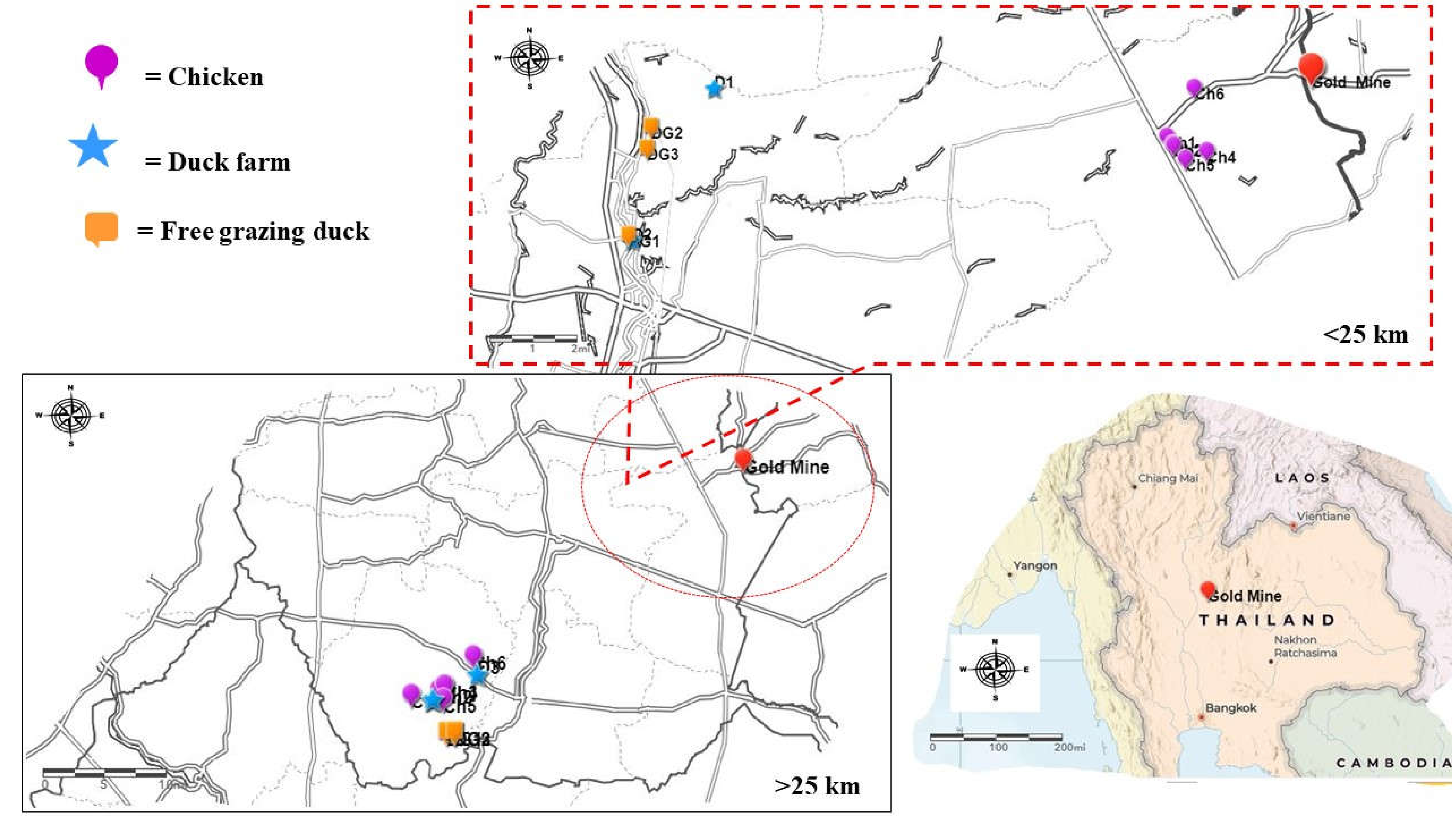

Figure S1. Map of the sampling locations for chicken, farm duck, and free-grazing duck by using the ArcGIS program.

Table S1. Descriptive data collected using questionnaires at poultry farms; (-) means no sample.

Items	<25 km area			>25 km area		
	Chicken	Duck farm	Free-grazing duck	Chicken	Duck farm	Free-grazing farm
Total number of farms	6	2	3	6	3	3
Total number of animals	11-120	3,500-4,000	6,000	18-50	2,000-10,000	1,500-5,000
**Chicken breed**						
Rhode Island red	100% (6/6)	-	-	100% (6/6)	-	-
**Duck breed**						
Khaki Campbell	-	100% (2/2)	100% (3/3)	-	66.66% (2/3)	100% (3/3)
Khaki Campbell mixed	-	-	-	-	33.33% (1/3)	-
Age of animal sampled (months)	12.92	10.50	11.5	7.83	6.00	6.66
**Poultry feed**						
Commercial feeds	50% (3/6)	50% (1/2)	-	-	33.33% (1/3)	-
Self-Mixed feeds	-	50% (1/2)	-	-	-	-
Commercial and Self-Mixed feeds	50% (3/6)	-	-	100% (6/6)	66.66 (2/3)	-
Grazing naturally	-	-	100% (3/3)	-	-	100% (3/3)
**Drinking water**						
Tap water	66.66% (4/6)	-	-	83.33% (5/6)	66.66% (2/3)	-
Groundwater	-	-	-	-	33.33% (1/3)	-
Tap water and groundwater	16.66% (1/6)	-	-	-	-	-
Canal water	16.66% (1/6)	100% (2/2)	100% (3/3)	16.66% (1/6)	-	100% (3/3)
**Farm location**						
Near the source of	16.66% (1/6)	100%(2/2)	100%(3/3)	-	100% (3/3)	100% (3/3)
agriculture in	83.33% (5/6)	-	-	100% (6/6)	-	-
community resources						
**Farm soil dredging**						
Yes	66.66% (4/6)	100% (2/2)	-	83.33% (5/6)	100% (3/3)	-
No	33.33% (2/6)	-	-	16.66% (1/6)	-	-
Utilization of manure						
Make household	66.66% (4/6)	100% (2/2)	-	83.33% (5/6)	66.66% (2/3)	-
fertilizers	33.33% (2/6)	-	-	16.66% (1/6)	33.33% (1/3)	-
No						
